# Multi-channel multiphase CT-based deep learning and radiomics fusion model for noninvasive pathological grading of clear cell renal cell carcinoma

**DOI:** 10.3389/fonc.2025.1710329

**Published:** 2026-01-15

**Authors:** Chongyang Sun, Qi Chen, Meng Gao, Shiqi He, Ze Zhang, Wei Zhang, Xigang Xiao

**Affiliations:** 1 CT Department of First Affiliated Hospital of Harbin Medical University, Harbin, China; 2 Ultrasound Department of First Affiliated Hospital of Harbin Medical University, Harbin, China; 3 Urology Department Ward 1 of First Affiliated Hospital of Harbin Medical University, Harbin, China; 4 Graduate Department of First Affiliated Hospital of Harbin Medical University, Harbin, China

**Keywords:** ccRCC, CT, deep learning, multi-channel, pathological grading

## Abstract

**Purposes:**

To develop a combined model integrating multi-channel deep learning features, radiomics features, and clinical variables for noninvasive pathological grading of clear cell renal cell carcinoma (ccRCC).

**Material and methods:**

A retrospective study was conducted on 496 patients with pathologically confirmed ccRCC who underwent preoperative triple-phase contrast-enhanced CT. Multi-channel deep learning features were extracted from three ROI settings (conventional, tumor-only, and 5-mm expansion) by stacking arterial, medullary and excretory phases. These were fused with arterial-phase radiomics features and clinical data to construct and compare predictive models.

**Results:**

In the ResNet50 model, the expanded 5mm ROI slice model had an AUC of 0.791 in the training cohort and 0.780 in the testing cohort, indicating that the model could effectively predict the pathological grading of ccRCC. By combining deep learning features with radiomics and clinical features, the integrated model achieved AUCs of 0.855 in the training cohort and 0.849 in the testing cohort, significantly outperforming the individual radiomics and clinical models. Decision curve analysis (DCA) further showed that the clinical-imaging combined model provided a higher net benefit.

**Conclusion:**

Multi-channel, multiphase CT fusion, when integrated with radiomics and clinical features, can significantly enhance predictive accuracy for ccRCC grading, providing a promising and interpretable noninvasive tool to support individualized treatment planning.

## Introduction

Clear cell renal cell carcinoma (ccRCC) is the most common subtype of renal malignancy, accounting for approximately 70–80% of all renal cell carcinoma cases (1, 2). Pathological grading of ccRCC plays a pivotal role in assessing tumor aggressiveness, metastatic potential, and patient prognosis (3–5). Conventional grading methods rely on histopathological biopsy. However, due to the pronounced spatial and temporal heterogeneity of ccRCC, a single biopsy specimen often fails to fully capture the tumor’s biological characteristics, thereby limiting the accuracy of treatment planning (6, 7). This limitation underscores the urgent need for novel diagnostic strategies that can improve the accuracy and reliability of pathological grading (8).

In recent years, imaging-based, noninvasive predictive models have emerged as promising tools for tumor grading, risk stratification, and treatment guidance (9–11). Among these, radiomics has attracted considerable attention by enabling the extraction of high-throughput quantitative features from standard-of-care medical images, revealing patterns beyond visual perception (12). These handcrafted features—covering intensity, texture, and shape—can be used to train machine learning algorithms for disease classification and prognosis prediction (13). However, the discriminative power of radiomics is often constrained by the predefinition of feature sets and susceptibility to variations in acquisition protocols (14, 15).

Deep learning, which simulates the cognitive processes of the human brain by constructing multi-layer neural networks, optimizes a vast number of parameters via the backpropagation algorithm and progressively extracts features through nonlinear activation functions, has achieved remarkable success in medical imaging (16). Its powerful feature-learning capability enables end-to-end modeling without the need for handcrafted feature engineering, allowing for the automatic discovery of complex hierarchical representations from raw data (17). Convolutional neural networks (CNNs), in particular, have been extensively applied in tasks such as tumor detection, segmentation, and grading across various organ systems, demonstrating robustness and generalizability when adequately trained on diverse datasets (18–20).

In the development of deep learning models for tumor grading, different regions of interest (ROIs) allow networks to capture tumor biological characteristics at multiple spatial scales (21–23). Narrow ROIs focus on the lesion core, potentially highlighting intra-tumoral heterogeneity, whereas expanded ROIs may include peritumoral tissue, which can harbor biologically relevant cues such as edema, angiogenesis, or subtle infiltration. By comparing model performance across different ROI configurations, one can evaluate the impact of spatial context on pathological grading accuracy and determine the optimal imaging analysis strategy.

Multi-channel deep learning represents a cutting-edge paradigm for cross-modal information integration, implemented through a parallel heterogeneous data-stream processing architecture (24). Its core concept involves employing independent branch networks to extract modality-specific features, followed by a hierarchical fusion strategy to construct a complementary and enriched joint representation (25). In the context of multiphasic contrast-enhanced CT, stacking arterial, medullary, and excretory phases as multi-channel inputs can enrich the feature space by simultaneously capturing tumor vascularity, parenchymal enhancement patterns, and delayed washout characteristics (26). Such fusion may enhance diagnostic accuracy, especially in conditions like ccRCC where vascular dynamics and tissue heterogeneity are key pathological hallmarks (27, 28).

Previous studies have attempted to apply artificial intelligence–based approaches for pathological grading of ccRCC; however, most of these efforts have remained limited to single-phase imaging or conventional radiomics analyses. such as those by Ki Choon Sim et al. (29) and Enming Cui et al. (30), extracted radiomics features independently from MRI and CT sequences and performed feature-level fusion, without prior image-level fusion. While effective, these approaches may underutilize the synergistic potential of spatially aligned, multi-phase image data. Gurbani et al. (31)developed a predictive model using three-dimensional radiomic features extracted from non-contrast and portal venous–phase CT images in combination with machine-learning methods, but the reported AUC ranged only from 0.58 to 0.69. This modest performance suggests that simple linear fusion of multiphase radiomic features is insufficient to fully capture the complex pathological heterogeneity of ccRCC. Such limitations are largely attributable to reliance on manually predefined features, inadequate utilization of spatial contextual information, and the absence of deep hierarchical feature learning. Building upon these prior efforts, the present study introduces several targeted methodological improvements. First, arterial-, medullary-, and excretory-phase contrast-enhanced CT images were integrated to construct a truly multichannel input structure, enabling end-to-end deep feature learning directly at the image level. Second, a 5 mm peritumoral expansion was incorporated to capture critical information related to the tumor microenvironment and angiogenesis. Finally, deep-learning–derived features were fused with radiomic features and clinical variables to establish a multidimensional integrative prediction framework. This design aims to overcome the limitations of previous studies related to single-phase imaging, manually predefined features, and insufficient exploitation of spatial information, thereby enabling a more accurate and comprehensive noninvasive assessment of ccRCC pathological grading.

Accordingly, this study seeks to develop a genuinely multichannel, multiphase deep-learning–based fusion model that integrates radiomics and clinical information, providing an interpretable and clinically applicable noninvasive tool for pathological grading of ccRCC. We hypothesize that such integration will enable efficient, noninvasive pathological grading of ccRCC, ultimately facilitating more precise and individualized treatment strategies.

## Materials and methods

### Study population

**Figure 1:**
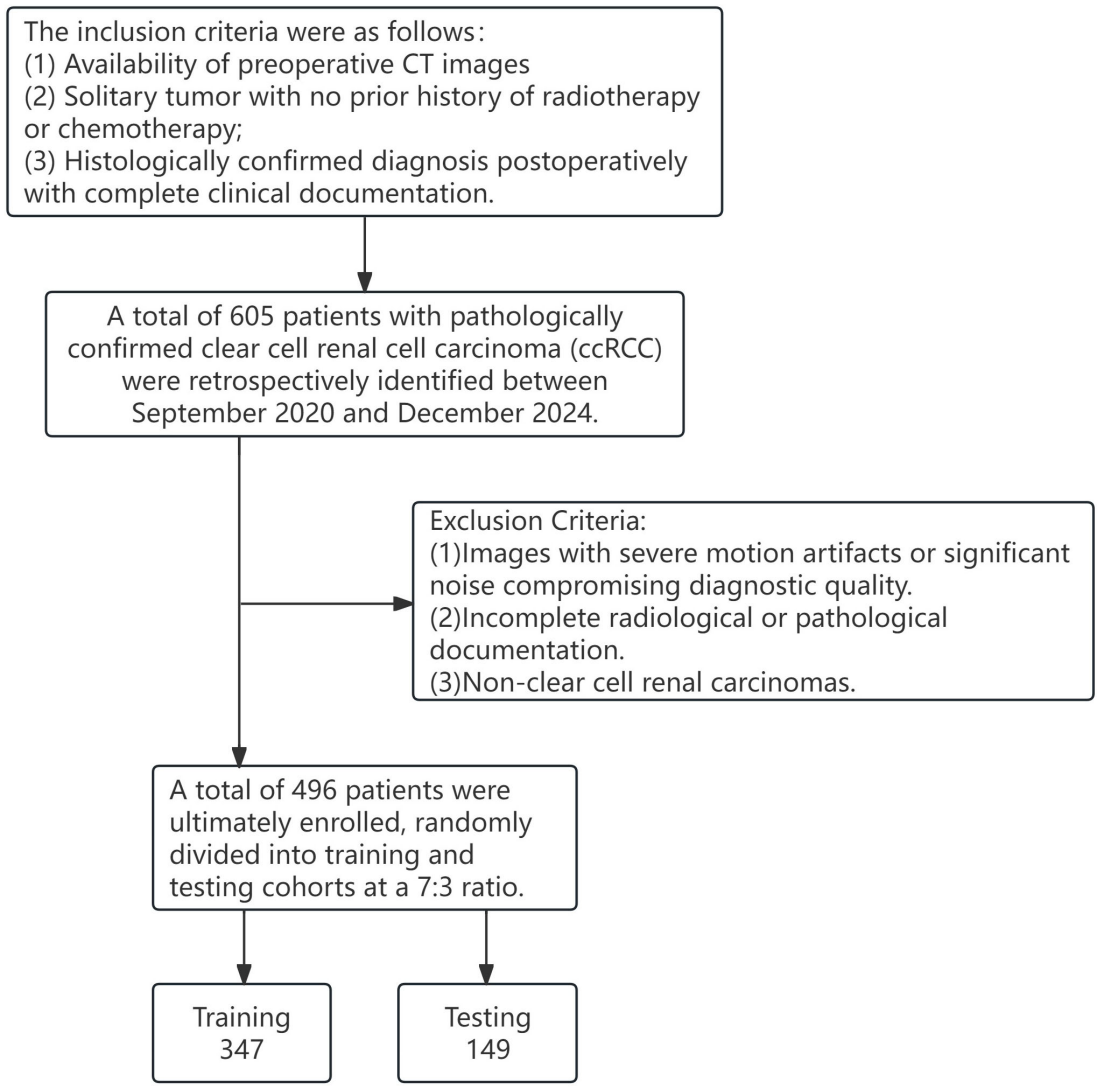
Study population enrollment process.

This retrospective study was approved by our institutional review board, and the requirement for informed consent was waived. All procedures were conducted in accordance with the Declaration of Helsinki. From June 2021 to December 2024, consecutive patients with pathologically confirmed clear cell renal cell carcinoma (ccRCC) who underwent preoperative contrast-enhanced CT scans at our institution were enrolled. Inclusion criteria were as follows: (1) Availability of preoperative triphasic contrast-enhanced CT images, including arterial, medullary, and excretory phase CT imaging data; (2) Solitary tumor with no prior history of radiotherapy or chemotherapy; (3) Histologically confirmed diagnosis postoperatively with complete clinical documentation. Exclusion criteria were: (1)Images with severe motion artifacts or significant noise compromising diagnostic quality.(2) Incomplete radiological or pathological documentation.(3)Non-clear cell renal carcinomas. All cases were randomly divided into a training cohort (70%; n = 347) and a testing cohort (30%; n = 149). The split was performed in Python using a fixed random seed 2025 to ensure reproducibility. No stratified sampling was applied; cases were allocated purely at random. Baseline characteristics were then compared between the two cohorts, with continuous variables assessed using the t-test and categorical variables evaluated using the chi-square test (**Figure 1**).

### Clinical and imaging data

Clinical variables including sex, age, hematuria, and flank pain were collected from the hospital information system (HIS). Pathological grading was assessed according to the WHO/ISUP grading system, which classifies tumors into four grades; grades 1–2 were defined as low grade and grades 3–4 as high grade. Two radiologists (with 6 and 8 years of CT experience, respectively) independently reviewed preoperative triple-phase CT scans to assess imaging features, including presence of calcification, intratumoral necrosis, and tumor interlobular arteries during the arterial phase. Both readers were blinded to the pathological diagnosis. Each tumor was assessed once by one radiologist; discrepancies were resolved by consensus with a third senior radiologist.

### CT acquisition protocol

All CT examinations were performed using high-end multi-detector CT scanners (Philips Brilliance iCT 256, GE Revolution 128). Triple-phase contrast-enhanced scans included the arterial, medullary, and excretory phases. Scanning parameters were: tube voltage, 120 kV; tube current, 250 mA; slice thickness, 1 mm(Philips Brilliance iCT 256),1.25mm(GE Revolution 128); and interslice gap, 1 mm(Philips Brilliance iCT 256), 1.25mm(GE Revolution 128). A nonionic iodinated contrast agent (70 mL) was injected at 4 mL/s, followed by a saline flush. The scan range covered the entire kidneys and tumors to ensure complete lesion capture.

### Image segmentation

All CT data were resampled to an isotropic voxel size of 1 mm×1 mm×1 mm to reduce inter-scanner variability. Window level and width were standardized to 50 HU and 350 HU, respectively, and intensity normalization was performed using Z-score standardization. Two experienced radiologists manually delineated three-dimensional regions of interest (ROIs) for each tumor in the arterial, medullary, and excretory phases using ITK-SNAP v3.8.0 software. Discrepancies were resolved by a third senior radiologist. To assess interobserver reproducibility, 50 randomly selected cases were used to calculate the intraclass correlation coefficient (ICC), with ICC > 0.75 indicating good agreement.

### 2D ROI construction and multi-channel deep learning model development

Tumor ROIs were extracted from the original images using ITK-SNAP software. All tumors were manually segmented on three-dimensional CT images to ensure accurate and reproducible ROI delineation. On this basis, to construct the deep-learning model while balancing computational efficiency and input standardization, each three-dimensional segmentation was reviewed slice by slice along the axial direction, and the slice with the largest tumor cross-sectional area was selected as the representative two-dimensional input. This slice typically contains the richest histological heterogeneity and morphological features and is considered to reasonably reflect the overall biological characteristics of the tumor; therefore, it has been widely adopted in imaging analyses of renal cell carcinoma and other solid tumors. To evaluate the robustness of the model with respect to slice selection, a prediction consistency analysis was performed on adjacent slices in randomly selected cases. The preliminary results indicated only minimal variation in model predictions when adjacent slices were used as inputs, suggesting a certain degree of stability with regard to two-dimensional slice selection. This strategy helps ensure that model performance does not rely on a single slice input, thereby enhancing its reproducibility and reliability in practical applications. Three ROI configurations were generated: Original ROI slice, encompassing the entire kidney region, providing a global context for tumor analysis; Tumor-only ROI slice, focusing solely on the tumor core to reduce background noise; Expanded ROI slice (Enlarge 5mm peritumoral region), incorporating peritumoral tissues to capture tumor microenvironmental features. All images were resized to 224×224 pixels. For each case, ROIs from the three contrast phases were stacked along the channel dimension to form a 224×224×3 multi-channel image as input.

**Figure 2:**
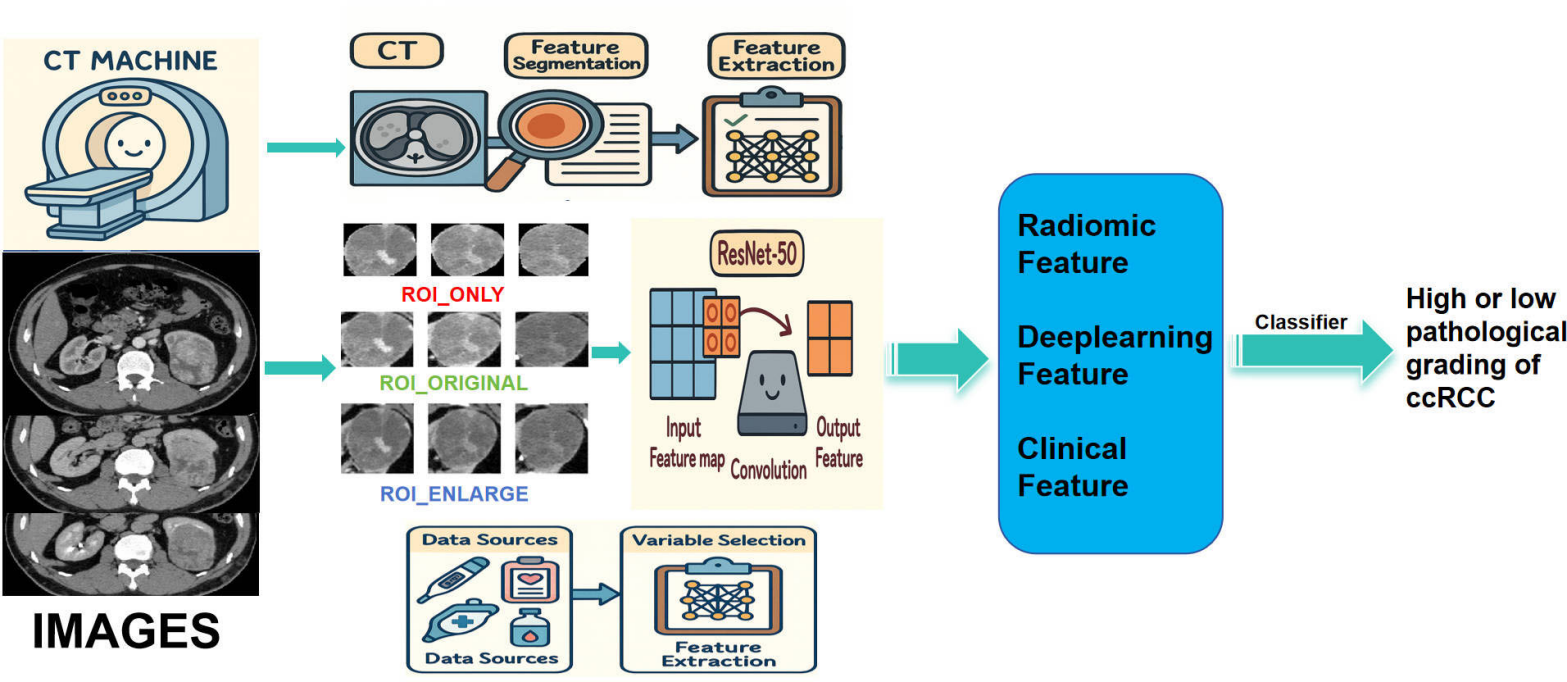
Workflow of this study.

A ResNet50-based transfer learning framework was employed. Model training involved backpropagation for parameter optimization, with real-time data augmentation (random horizontal flipping and cropping). The cross-entropy loss function was used, with an initial learning rate of 0.01, cosine annealing schedule, 50 training epochs, and a batch size of 128. After training, deep learning features were extracted from the penultimate global average pooling layer for each ROI configuration. These features were then combined with radiomics features to construct and compare models (**Figure 2**).

Model interpretability was evaluated using gradient-weighted class activation mapping (Grad-CAM), which generates heatmaps highlighting regions contributing most to classification decisions.

### Radiomics-deep learning model construction

Based on previous evidence that arterial-phase CT features are highly discriminative for ccRCC grading, radiomics features were extracted only from arterial-phase images (30, 32). The arterial phase is more sensitive to variations in tumor perfusion and enhancement heterogeneity, thereby providing more discriminative information related to vascular supply and microvascular characteristics. Meanwhile, our deep learning branch integrates tri-phasic multi-channel images at the image level, capturing the complementary enhancement patterns across phases. Therefore, we chose arterial-phase radiomics as a relatively independent, complementary source of information, which helps maximize informational gain while avoiding repetitive multi-phase radiomic inputs. This design reduces feature redundancy and the risk of overfitting, and ultimately improves the robustness and interpretability of the fusion framework. These included first-order statistics, shape features, and multiple texture descriptors. Deep learning features from the three ROI configurations were fused separately with arterial-phase radiomics features to form: comb-1: tumor-only ROI deep learning features + arterial-phase radiomics features; comb-2: conventional ROI deep learning features + arterial-phase radiomics features; comb-3: expanded ROI deep learning features + arterial-phase radiomics features. In the training set, feature selection was performed in two steps: Analysis of variance (ANOVA) to retain features with ICC > 0.9 and statistically significant differences; Least absolute shrinkage and selection operator (LASSO) regression with 10-fold cross-testing to identify features with non-zero coefficients. logistic regression (LR), support vector machine (SVM), and random forest (RF) were evaluated. Diagnostic performance was compared using the area under the curve (AUC), accuracy, sensitivity, specificity, positive predictive value (PPV), and negative predictive value (NPV), and the best-performing radiomics model was selected.

### Clinical-radiomics-deep learning model integration

To enhance clinical utility, the optimal radiomics–deep learning model was integrated with clinical variables to build a combined model. Model performance was assessed in both training and testing cohorts using AUC, accuracy, sensitivity, specificity, PPV, and NPV. Differences in AUCs between models were compared using the DeLong test to assess the statistical significance of model discriminative performance. Model calibration was evaluated by plotting calibration curves, which compare predicted probabilities with observed outcomes, thereby reflecting the reliability of the model in risk prediction. Decision curve analysis (DCA) was conducted to evaluate net clinical benefit across a range of threshold probabilities, thereby assessing the potential clinical impact in ccRCC grading (33).

### Statistical analysis

Categorical variables were compared using the chi-square test or Fisher’s exact test, as appropriate. Continuous variables were compared using the Mann-Whitney U test or the independent-samples t test. Model performance was evaluated using the area under the receiver operating characteristic curve (AUC), accuracy, sensitivity, and specificity. The 95% confidence interval (CI) for the AUC was calculated using the AUC function in the pROC R package. Differences in AUCs were compared using DeLong’s test, with P < 0.05 considered statistically significant. All analyses were performed using OnekeyAI, R software (version 4.3.3) and Python (version 3.11.11).

## Results

### Patient characteristics and clinical features

A total of 496 patients were included in this study. The clinical information and imaging characteristics of the patients are summarized in **Table 1**. In the training cohort, there were 227 males and 120 females, while the testing cohort included 93 males and 56 females. The mean age was 60.89 ± 10.06 years in the training cohort and 59.28 ± 9.72 years in the testing cohort. Among the clinical variables, hematuria showed a statistically significant difference between the training and testing cohorts (P < 0.05). Among the imaging features, intratumoral necrosis and the presence of tumor interlobular arteries were significantly different between the two cohorts (P < 0.05). No significant differences were observed for age and calcification in the testing cohort, for sex in the training cohort, or for flank pain in either cohort. Univariate and multivariate analyses revealed that only age was significantly associated with pathological grade, while the remaining variables showed no significant associations (**Table 2**). A clinical model was subsequently developed based on these findings.

**Table 1:** Characteristics of patients with ccRCC in the training and test cohorts.

Teature_Name	Train-label=ALL	Train-label=0	Train-label=1	p value	Test-label=ALL	Test-label=0	Test-label=1	P value
Age	60.89±10.06	59.64±10.64	63.10±8.56	0.011	59.28±9.72	58.43±9.98	60.73±9.15	0.164
Gender				0.381				0.004
0	227(65.42)	141(63.51)	86(68.80)		93(62.42)	50(53.19)	43(78.18)	
1	120(34.58)	81(36.49)	39(31.20)		56(37.58)	44(46.81)	12(21.82)	
Hematuria				<0.001				0.002
0	306(88.18)	206(92.79)	100(80.00)		125(83.89)	86(91.49)	39(70.91)	
1	41(11.82)	16(7.21)	25(20.00)		24(16.11)	8(8.51)	16(29.09)	
Lumbago				0.295				0.517
0	241(69.45)	159(71.62)	82(65.60)		101(67.79)	66(70.21)	35(63.64)	
1	106(30.55)	63(28.38)	43(34.40)		48(32.21)	28(29.79)	20(36.36)	
Calcification				0.003				0.121
0	299(86.17)	201(90.54)	98(78.40)		129(86.58)	85(90.43)	44(80.00)	
1	48(13.83)	21(9.46)	27(21.60)		20(13.42)	9(9.57)	11(20.00)	
Necrosis				0.003				0.004
0	101(29.11)	77(34.68)	24(19.20)		53(35.57)	42(44.68)	11(20.00)	
1	246(70.89)	145(65.32)	101(80.80)		96(64.43)	52(55.32)	44(80.00)	
Intertumoral_artery				0.002				<0.001
0	190(54.76)	136(61.26)	54(43.20)		88(59.06)	75(79.79)	13(23.64)	
1	157(45.24)	86(38.74)	71(56.80)		61(40.94)	19(20.21)	42(76.36)	

In the gender variable, 0 represents male and 1 represents female; for the other features, 0 indicates ‘no’ and 1 indicates ‘yes’.

**Table 2:** Univariate and multivariable logistic regression analysis for clinical features.

feature_name	OR_UNI	OR lower 95%CI_UNI	OR upper 95%CI_UNI	p_value_UNI	OR_MULTI	OR lower 95%CI_MULTI	OR upper 95%CI_MULTI	p_value_MULTI
Gender	0.481	0.350	0.664	0.001	0.722	0.488	1.067	0.171
Lumbago	0.683	0.493	0.945	0.054				
Necrosis	0.697	0.563	0.862	0.005	1.616	1.077	2.425	0.052
Intertumoral_artery	0.826	0.634	1.075	0.232				
Age	0.992	0.989	0.995	0.001	0.988	0.982	0.994	0.001
Calcification	1.286	0.797	2.075	0.388				
Hematuria	1.563	0.923	2.646	0.163				

### Performance and visualization of the deep learning model

**Figure 3:**
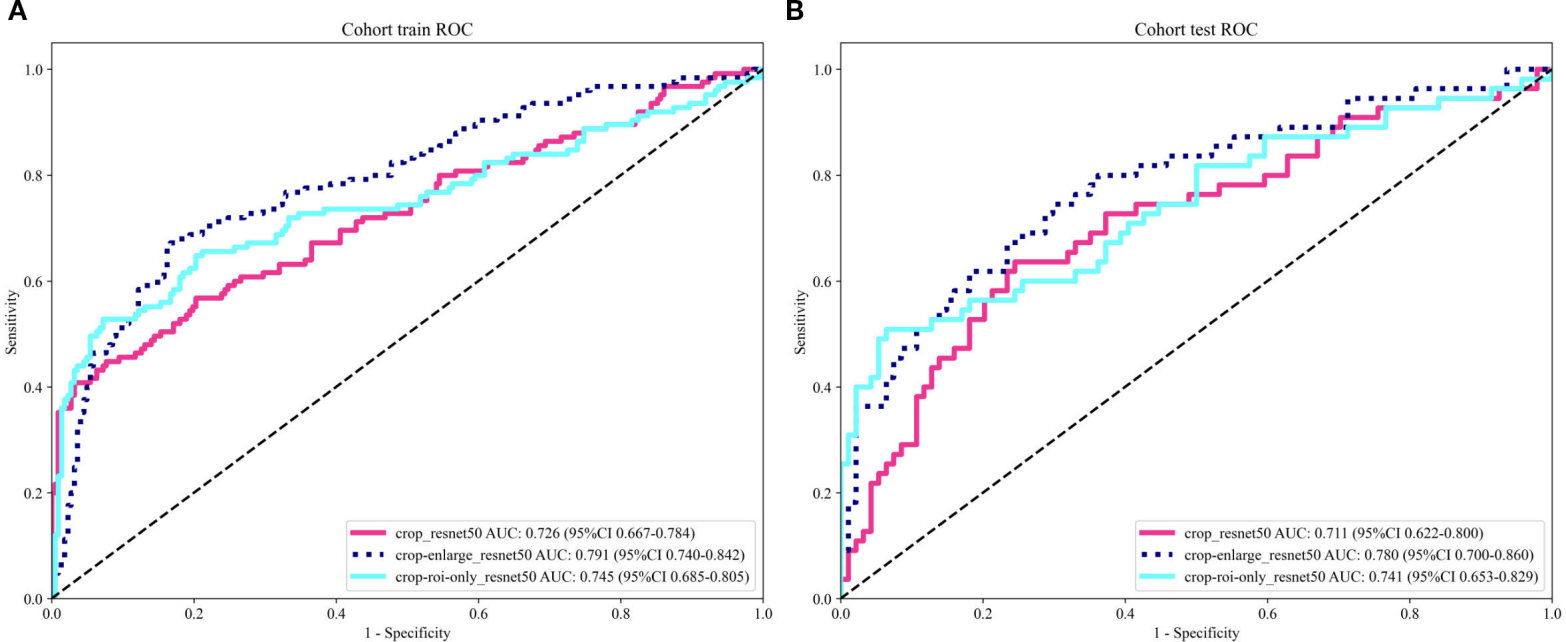
**(A, B)** The receiver operating characteristic (ROC) curves of three different ROI deep learning model in the training cohort and testing cohort.

ResNet50 demonstrates excellent stability and robustness in the development of medical imaging diagnostic models; therefore, this architecture was adopted for our deep learning analysis. As shown in **Table 3** and **Figures 3A, B**, among the different ROI configurations, the 5mm enlarged ROI achieved the best predictive performance. In the training cohort, the AUC was 0.791 (95% CI: 0.740–0.842), with an accuracy of 0.775, sensitivity of 0.772, specificity of 0.833, PPV of 0.694, and NPV of 0.819. In the testing cohort, the AUC was 0.780 (95% CI: 0.700–0.859), with an accuracy of 0.718, sensitivity of 0.745, specificity of 0.702, PPV of 0.594, and NPV of 0.825.

**Table 3:** The performance of three different ROI deep learning model in distinguishing the pathological grade of clear cell renal cell carcinoma.

Signature	Accuracy	AUC	95% CI	Sensitivity	Specificity	PPV	NPV	Cohort
crop	0.767	0.726	0.6667 - 0.7844	0.608	0.768	0.879	0.744	train
crop-enlarge	0.775	0.791	0.7401 - 0.8424	0.772	0.833	0.694	0.819	train
crop-roi-only	0.784	0.745	0.6853 - 0.8050	0.628	0.828	0.805	0.777	train
crop	0.711	0.711	0.6221 - 0.7999	0.636	0.755	0.603	0.78	test
crop-enlarge	0.718	0.78	0.7004 - 0.8598	0.745	0.702	0.594	0.825	test
crop-roi-only	0.779	0.741	0.6527 - 0.8286	0.709	0.836	0.824	0.765	test

**Figure 4:**
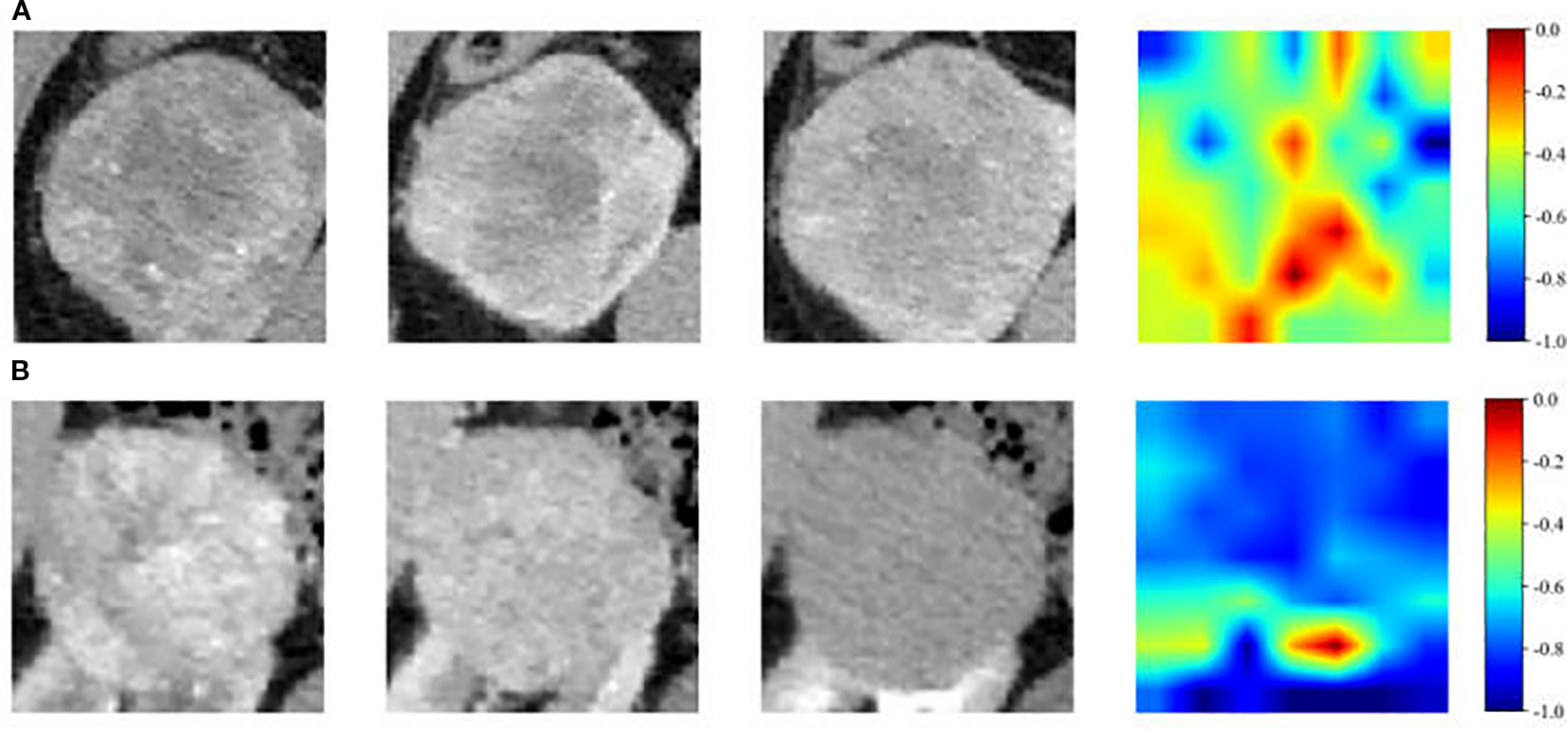
Representative images and illustration of the ccRCC pathological grade prediction visualization. **(A)** Feature map extracted by high-grade on CT image. Grad-CAM plots highlighted large, multiple regions activated within the tumor of patients. **(B)** Feature map extracted by low-grade on CT image. Grad-CAM plots emphasized very few regions activated within the tumor of patients.

Model interpretability was assessed using gradient-weighted class activation mapping (Grad-CAM), which visualized the spatial distribution of pixel importance in different colors, highlighting differences between high-grade and low-grade ccRCC tumors. Representative cases from the training cohort were examined. High-grade tumors generally exhibited larger activation regions, while low-grade tumors showed more limited activation. In most cases, the network demonstrated sensitivity to intratumoral heterogeneity and tumor margins in high-grade lesions (**Figure 4**).

### Comparison of combined model performance

Deep learning features were further extracted from the trained models and fused with arterial-phase radiomics features to construct combined models. After LASSO feature selection, the three combined models retained 24, 31, and 34 non-zero coefficient features, respectively (**Supplementary Figures S1A–I**). As shown in **Supplementary Figures S2A–F** and **Supplementary Table S1**, although the conventional ROI slice based combined model achieved good performance in the training cohort, it showed lower AUC in the testing cohort, indicating a risk of overfitting. In contrast, the 5-mm expanded ROI slice–based combined model demonstrated better overall performance compared to the other two models.

**Figure 5:**
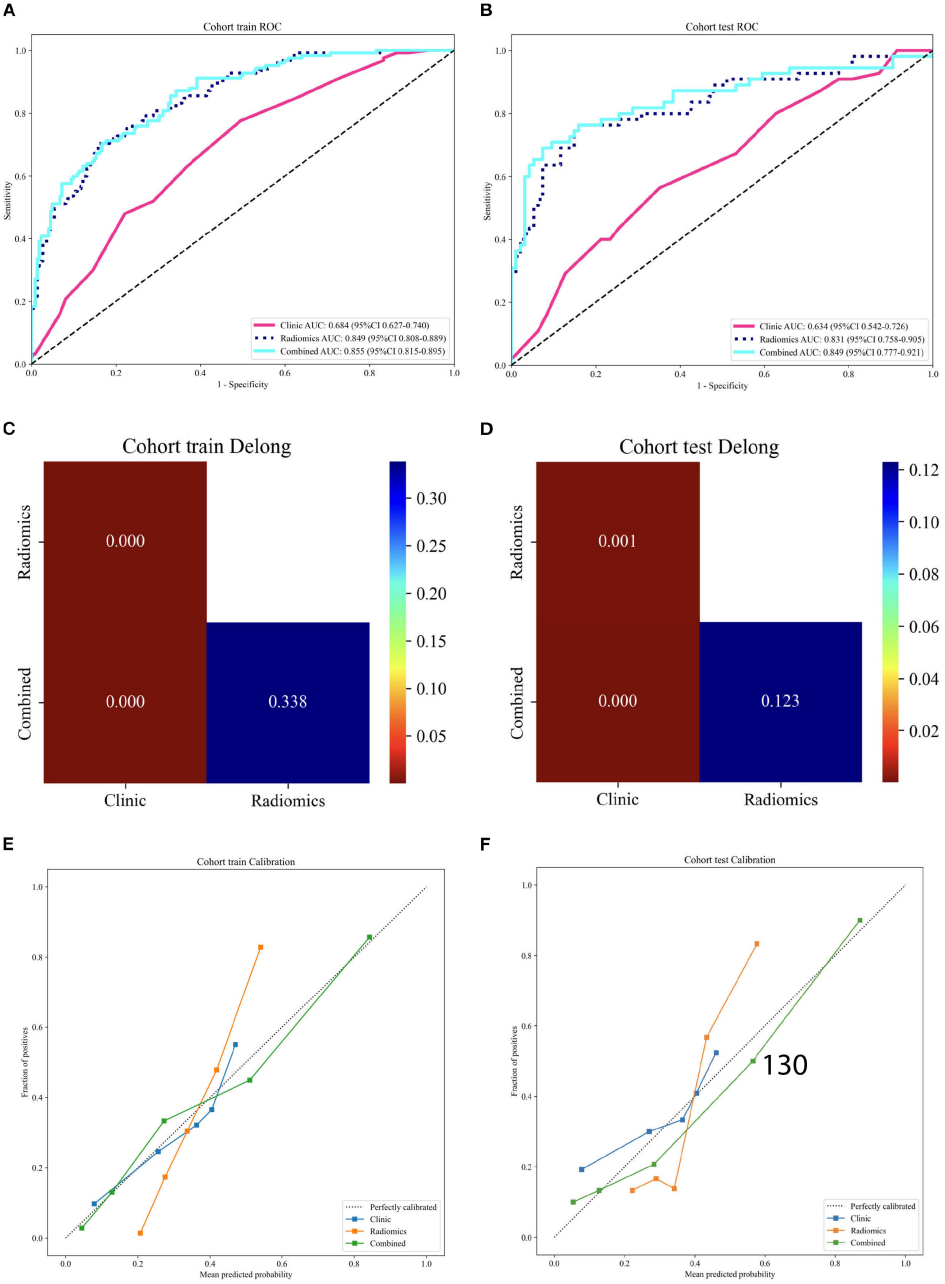
**(A, B)** The ROC curves of ROI-enlarge Clinical-imaging integrated model in the training cohort and testing cohort. **(C, D)** The DeLong’s test was used to compare the AUCs of the three models in the training cohort and testing cohort. **(E, F)** The calibration of the three models in the training cohort and testing cohort.

When comparing the three machine learning classifiers, logistic regression (LR), support vector machine (SVM), and random forest (RF). The RF classifier exhibited the most stable performance, with an AUC of 0.849 in the training cohort and 0.831 in the testing cohort. Although SVM achieved a higher AUC in the training cohort, its performance decreased in the testing cohort, suggesting potential overfitting. Therefore, the RF model was selected as the optimal classifier. Subsequently, imaging features from the optimal combined model were integrated with clinical variables to construct the clinical–imaging combined model (**Figures 5A, B**). In the training cohort, the combined model achieved an AUC of 0.855, and in the testing cohort, an AUC of 0.849. Its predictive ability was significantly higher than that of the clinical model alone and slightly better than the imaging combined model.

In the training cohort and testing cohort, DeLong’s test showed that both the radiomics model and the combined model significantly outperformed the clinical model (both p < 0.001), whereas no significant difference was observed between the combined and radiomics models (training:p = 0.338,testing: p = 0.123) (**Figures 5C, D**). In the calibration analysis of the training cohort and testing cohort, the combined model showed a calibration curve that was overall closer to the ideal reference line, indicating better agreement between predicted probabilities and the observed event rates. (**Figures 5E, F**).

**Figure 6:**
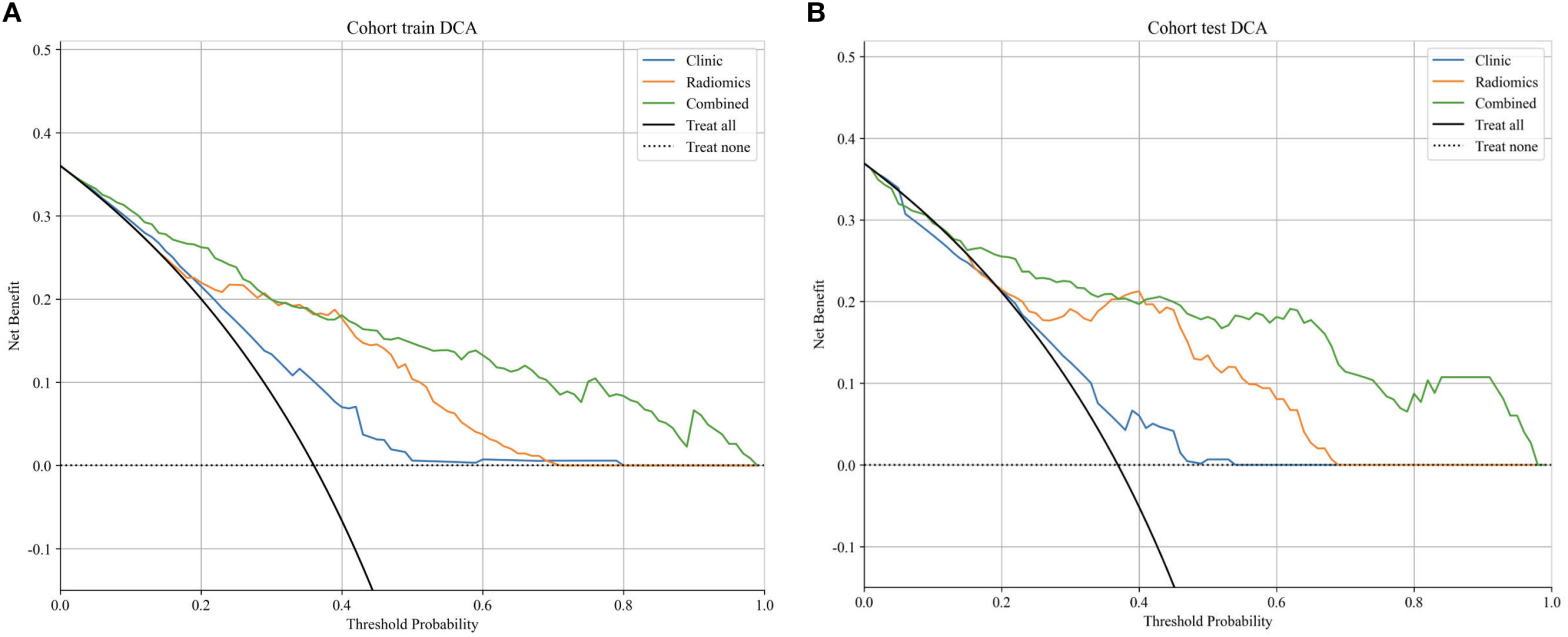
**(A, B)** Decision curve analysis (DCA) for three models in classifying the diagnostic pathological grading in training and test cohorts, respectively.

Decision curve analysis (DCA) demonstrated (**Figures 6A, B**) that the clinical-imaging combined model provided a higher net benefit across a range of threshold probabilities, indicating substantial potential for clinical application in ccRCC pathological grading.

## Discussion

Deep learning has demonstrated substantial advantages in multimodal fusion analysis within medical imaging (34–36). By integrating complementary information from CT, MRI, PET, and other imaging modalities, deep learning frameworks can characterize lesions from multiple perspectives, enabling more precise diagnosis and supporting clinical decision-making in complex cases (37–39). The ability to extract hierarchical, nonlinear features from heterogeneous data streams allows such models to capture subtle morphological and functional patterns that may be imperceptible to traditional analysis (40). As algorithms mature, datasets diversify, and clinical testing deepens, these approaches hold strong potential for advancing precision medicine and personalized treatment strategies (41–43).

In the present study, we developed a clinical-imaging combined model for ccRCC pathological grading by integrating deep learning features, radiomics features, and clinical variables. Compared with previous studies based on radiomics and traditional machine-learning approaches, the present study achieves substantial methodological advancements. Although prior multiphase imaging analyses—such as the dual-phase CT model reported by Gurbani et al. (31)—have improved the predictability of pathological grading to some extent, their performance remains constrained by hand-crafted feature design and single-level feature fusion, limiting the ability to fully exploit the multi-scale spatial information embedded within medical images. Our results demonstrated that the multi-channel model based on a 5-mm peritumoral expansion, when fused with radiomics features, achieved superior performance compared with models using conventional slices or tumor-only slices. The expanded ROI setting likely captures peritumoral microenvironmental cues, including subtle invasion patterns, microvascular proliferation, and perilesional heterogeneity, all of which are known to influence tumor aggressiveness. Furthermore, the expanded ROI reduces the risk of omitting informative peritumoral signals while simultaneously suppressing irrelevant background structures, thereby enhancing discriminative power. These findings underscore the importance of spatial context in noninvasive tumor grading.

Our work builds upon prior evidence supporting the utility of multi-dimensional imaging features for tumor classification. For example, Lin et al. (32) reported significant gains in renal tumor grading accuracy when combining radiomics with deep learning features. However, few previous studies have systematically compared multiple ROI ranges in conjunction with feature fusion, and even fewer have incorporated peritumoral expansion in a multi-phase CT setting. Yang et al. (44) and Xu et al. (45) demonstrated the predictive value of deep features extracted from contrast-enhanced imaging, particularly in fusion frameworks, but omitted the excretory CT phase. The excretory phase provides additional structural and functional information such as enhanced delineation of tumor parenchyma boundaries, assessment of delayed enhancement patterns, and indirect markers of renal function that can complement arterial and medullary phase data. By stacking arterial, medullary, and excretory phases into a three-channel input, our model captures a more comprehensive physiological and pathological profile, which proved beneficial for feature fusion and downstream classification.

Our findings also align with a recent systematic review comparing radiomics-only, deep learning-only, and fusion models, which reported that fusion models achieved the highest performance in 63% of included studies (46). Multi-channel imaging enables each channel to encode distinct aspects of tumor biology; when these channels are fused, the resulting feature space becomes richer and more robust to image noise, artifacts, or variability in a single phase. Moreover, this approach enhances the detection of subtle imaging patterns that may be overlooked by single-phase or handcrafted features alone. A key innovation of our approach lies in its dual-level integration: image-level fusion via multi-channel inputs and feature-level fusion with radiomics and clinical data. This hierarchical strategy enables the model to leverage low-level texture and intensity patterns while also incorporating handcrafted descriptors that may capture domain-specific cues. Compared with approaches relying solely on deep learning or radiomics, this hybrid method maximizes the complementary strengths of each while mitigating their individual limitations. The advantages of this design are further supported by our decision curve analysis, which demonstrated consistent net clinical benefit over a broad range of threshold probabilities, underscoring its potential for integration into routine diagnostic workflows.

From a clinical perspective, the integration of multiphasic CT-derived features with routine clinical data into a unified predictive framework is particularly appealing. Such models could serve as decision-support tools to identify high-risk patients, prioritize biopsy, or tailor surveillance intervals. Nevertheless, successful clinical adoption will require rigorous external testing across diverse populations and imaging systems, as well as the incorporation of interpretability tools capable of providing clinicians with both visual and quantitative explanations for model predictions. Future research could also investigate integration with complementary modalities such as MRI, histopathology, and genomics to further enrich the feature space and improve predictive accuracy.

It should be clarified that, although 3D tumor ROIs were used during the segmentation stage to accurately localize the tumor extent, the deep-learning branch adopted a slice-based 2.5D input strategy: for each phase, the single axial slice with the largest tumor cross-sectional area was selected, and the three-phase slices were fused at the image level as multi-channel inputs. The rationale for choosing the largest-area slice is that it typically contains more informative tumor morphology and enhancement heterogeneity patterns and is more reproducible than arbitrary slice selection, thereby achieving a balance between computational efficiency and informational content. Nevertheless, we acknowledge that the robustness of the model to slice selection has not been systematically quantified in the present study; in theory, alternative selection schemes may affect the degree to which intratumoral heterogeneity is captured, potentially leading to performance variability. Future work will therefore conduct controlled experiments to compare different slice selection strategies and, together with repeated resampling and cross-validation, further validate robustness to slice selection in multi-center external cohorts to improve clinical generalizability.

It is worth noting that the WHO/ISUP grading system inherently possesses a clear ordinal structure, in which grades from low to high reflect the progressive increase in nuclear atypia and biological aggressiveness of tumor cells. However, in the present study, to ensure model training stability and maintain comparability with prior studies, a binary classification strategy was adopted for pathological grading. While this approach simplifies the grading task, it inevitably overlooks the continuity and hierarchical progression between different grades. In future work, with expanded sample sizes and the incorporation of multicenter data, we plan to further explore multiclass classification models or ordinal regression frameworks to more precisely characterize the continuous pathological spectrum embodied in the WHO/ISUP grading system. By fully leveraging the inherent ordinal information among grades, such approaches may further improve the model’s discriminative performance for intermediate grades and enhance its applicability and interpretability in real-world clinical settings, thereby more comprehensively reflecting the biological continuity of ccRCC pathological grading.

We observed a modest shortfall in sensitivity. This may be attributable to limited sample size and class imbalance specifically, the underrepresentation of high-grade tumors leading to a bias toward majority-class (low-grade) predictions and thus more false negatives for aggressive disease (45). Previous literature has shown that such imbalance can substantially degrade machine learning performance, particularly in high-stakes clinical prediction tasks. Additional factors, including noise, artifacts, and inter-scanner resolution variability, can further challenge feature extraction and model generalization. To mitigate these issues, future work should consider advanced sampling strategies, synthetic minority oversampling, cost-sensitive or focal loss functions, and targeted augmentation to improve sensitivity for high-grade tumors. Moreover, incorporating harmonization algorithms to minimize scanner-related variability and integrating multi-center datasets could enhance the robustness of the model.

Several limitations warrant acknowledgment. First, this study was based on a single-center retrospective cohort. The case sources and imaging acquisition conditions were relatively consistent, which helped ensure data quality and analytical consistency to a certain extent. However, this study design may also introduce potential selection bias, as the samples were derived from a single institution with relatively homogeneous patient populations and scanning conditions. The model’s generalizability has not been fully validated across different institutions, scanners and reconstruction settings, or variations in imaging workflows and patient populations. Therefore, its performance may decline to some extent when applied in external real-world settings. Although a random split was used in this study, baseline imbalances may still occur when the sample size is limited and the event distribution is uneven, as random sampling can produce inter-cohort differences due to sampling variability. Future work will adopt more robust internal validation strategies, such as stratified random splitting and repeated resampling, to improve the reliability and generalizability of the results. Second, although our dataset was of reasonable size, expanding the cohort, particularly increasing the proportion of high-grade tumors would enable more balanced training and reduce variability in performance estimates. Third, while our fusion framework effectively integrated deep learning and radiomics features, the optimal strategies for feature selection, fusion, and hyperparameter tuning remain to be determined. Advanced interpretability methods beyond Grad-CAM, such as attention-based saliency mapping or SHAP value analysis, could yield deeper insights into model decision-making and facilitate its acceptance in clinical practice.

We will conduct external validation in multi-center cohorts encompassing different institutions, scanner vendors, and acquisition/reconstruction protocols to more rigorously assess the model’s generalizability. In parallel, we will explore more advanced fusion strategies—such as attention- or gate-based fusion methods and multimodal transformer architectures—to better exploit the complementary information across phases/modalities, reduce redundancy, and improve the robustness and interpretability of the framework.

In summary, we present a multi-channel, multiphase CT based deep learning and radiomics fusion model for noninvasive ccRCC grading. By incorporating peritumoral expansion and leveraging complementary phase information, our framework generates a more expressive and robust feature representation, achieving strong predictive performance while maintaining clinical interpretability. Despite certain limitations, the model holds considerable promise as a decision-support tool for risk stratification and treatment planning. Future work should focus on multi-institutional testing, integration with additional modalities, and exploration of real-time deployment within clinical workflows.

## Data Availability

The datasets presented in this article are not readily available. The raw data requires further research and cannot be uploaded to public websites. Requests to access the datasets should be directed to CS, 814154@hrbmu.edu.cn.

## Supplementary material

Supplementary Figure 1 **(A–I)**: Radiomics features’ results of comb-1、comb-2、comb-3.

Supplementary Figure 2 **(A, B)**: The ROC curves of ROI-only imaging-combined model in the training cohort and testing cohort. **(C, D)**: The ROC curves of ROI-original imaging-combined model in the training cohort and testing. **(E, F)**: The ROC curves of ROI-enlarge imaging-combined model in the training cohort and testing.

Supplementary Table 1 The performance of three image fusion model in distinguishing the pathological grade of clear cell renal cell carcinoma.
