# Supplementary figures and images for: Multi-channel multiphase CT-based deep learning and radiomics fusion model for noninvasive pathological grading of clear cell renal cell carcinoma
Source: Front Oncol. 2026 Jan 15;15:1710329. doi: 10.3389/fonc.2025.1710329 (PMC12851889; doi:10.3389/fonc.2025.1710329)

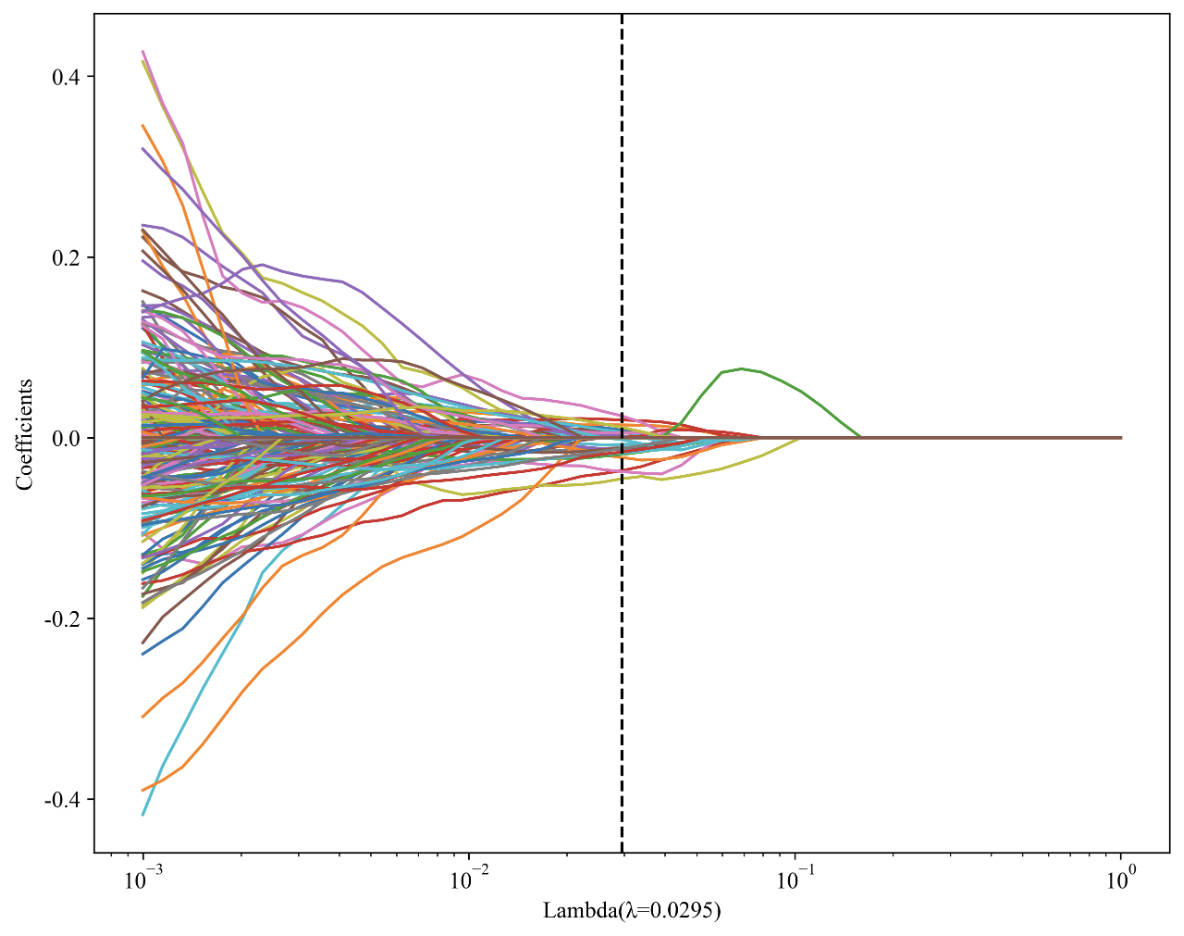

Supplement: Supplementary Figure 1 — (A–I): Radiomics features’ results of comb-1、comb-2、comb-3. [file Image1.jpeg]

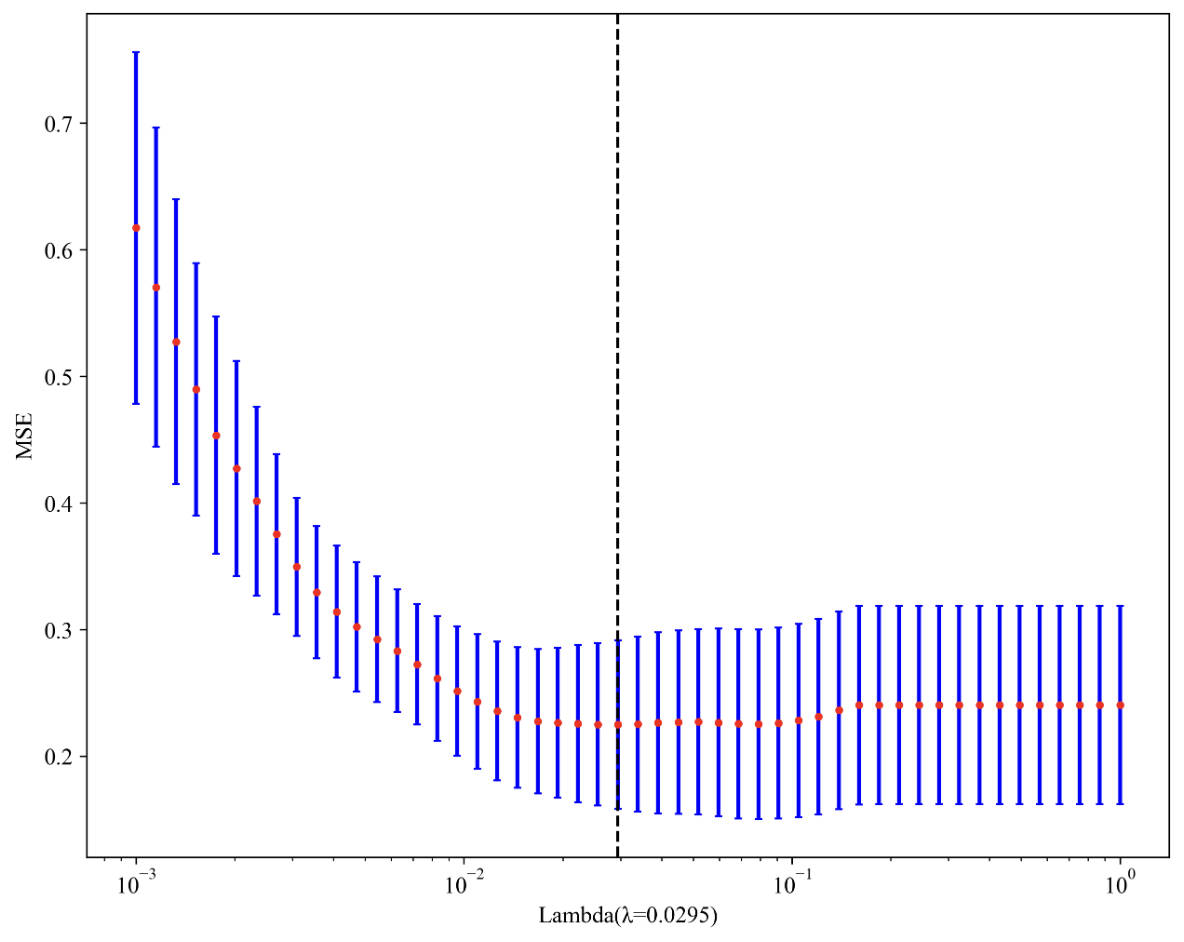

Supplement: Supplementary Figure 2 — (A, B): The ROC curves of ROI-only imaging-combined model in the training cohort and testing cohort. (C, D): The ROC curves of ROI-original imaging-combined model in the training cohort and testing. (E, F): The ROC curves of ROI-enlarge imaging-combined model in the training cohort and testing. [file Image2.jpeg]

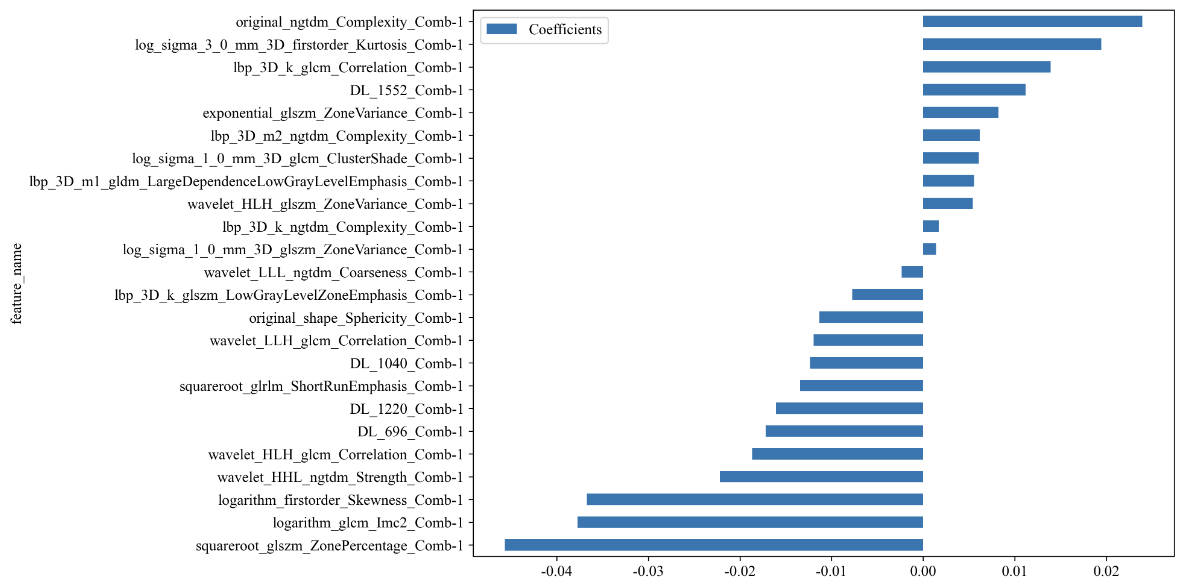

Supplement: Supplementary Table 1 — The performance of three image fusion model in distinguishing the pathological grade of clear cell renal cell carcinoma. [file Image3.jpeg]

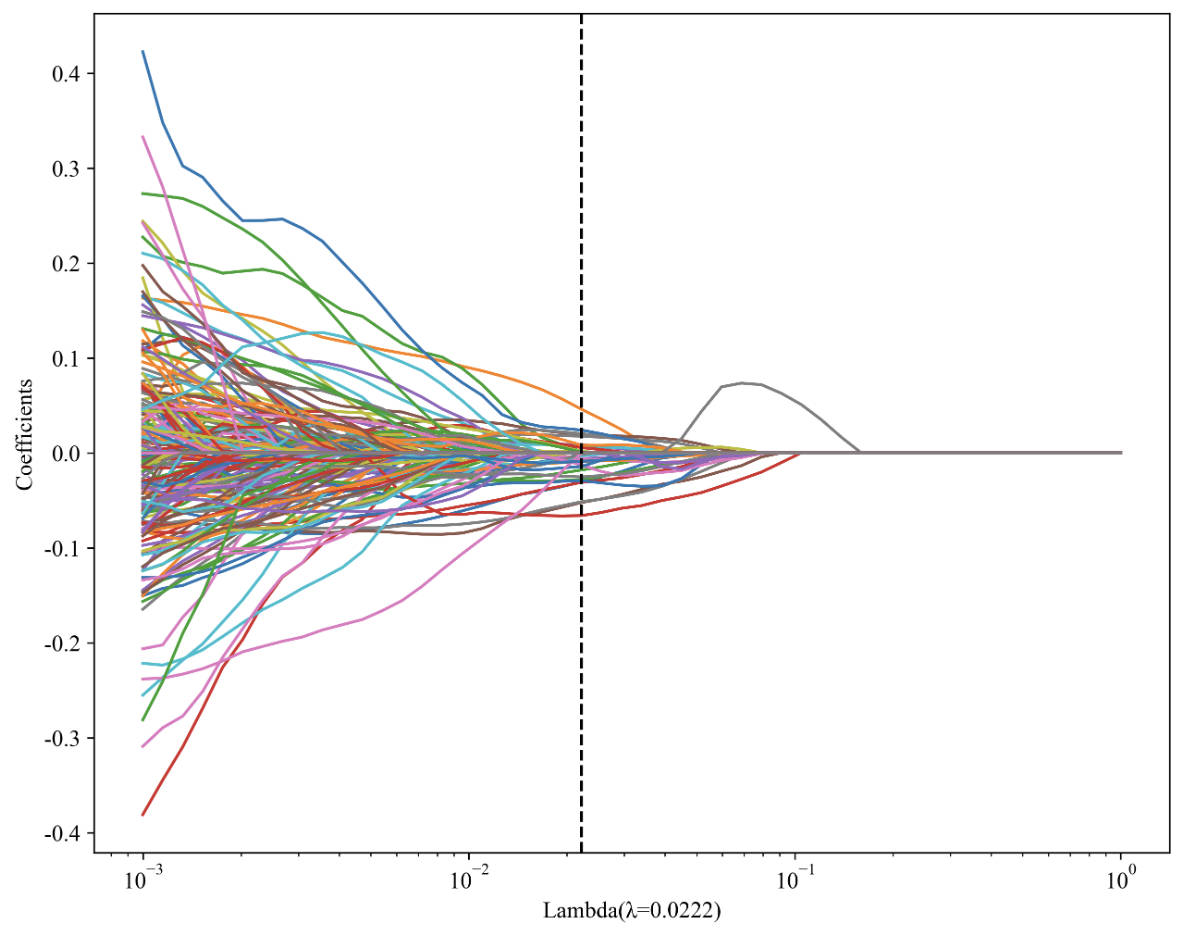

Supplement: Supplementary file 4 [file Image4.jpeg]

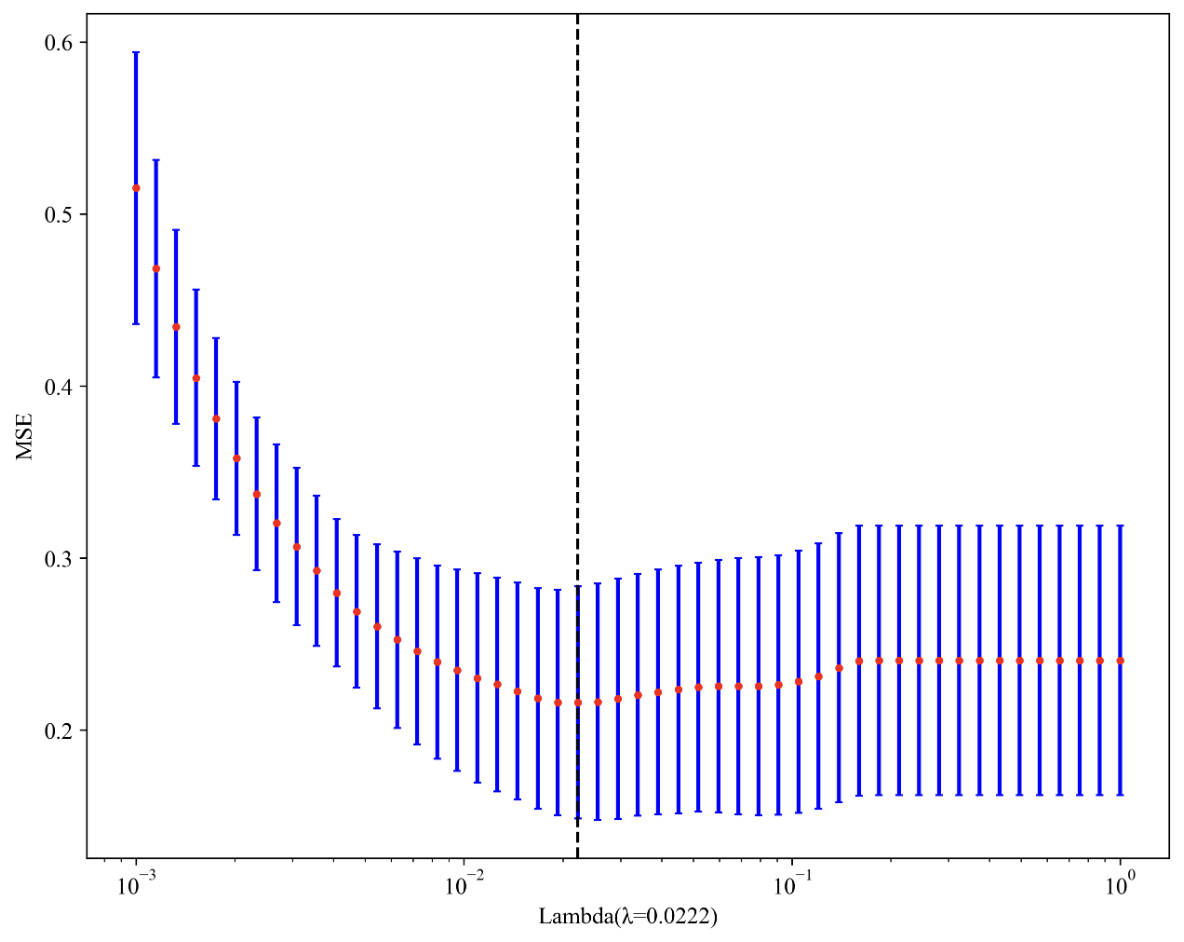

Supplement: Supplementary file 5 [file Image5.jpeg]

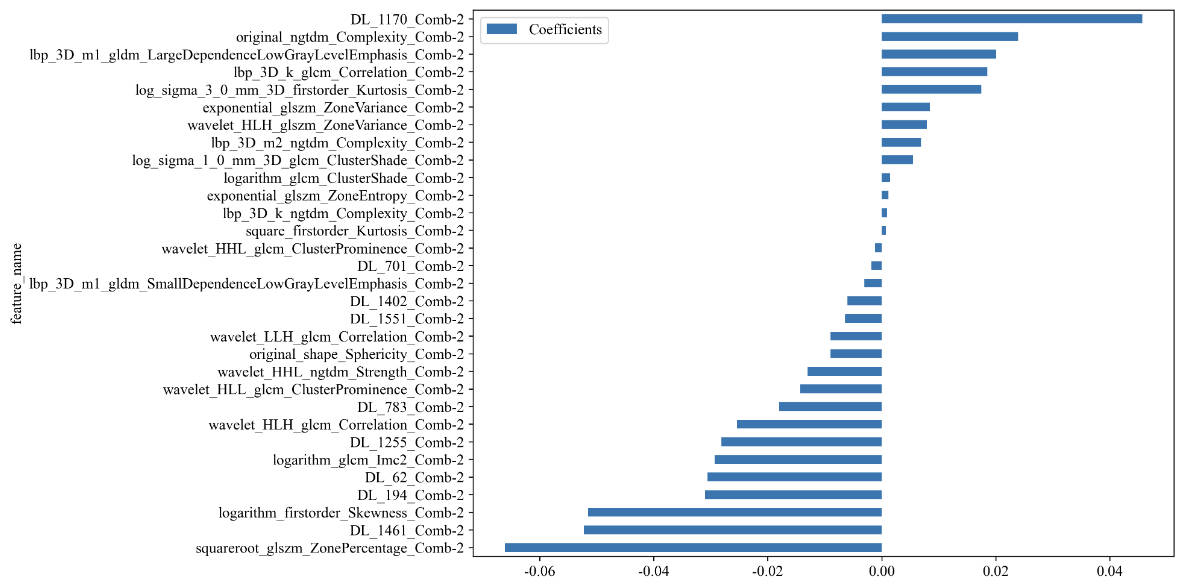

Supplement: Supplementary file 6 [file Image6.jpeg]

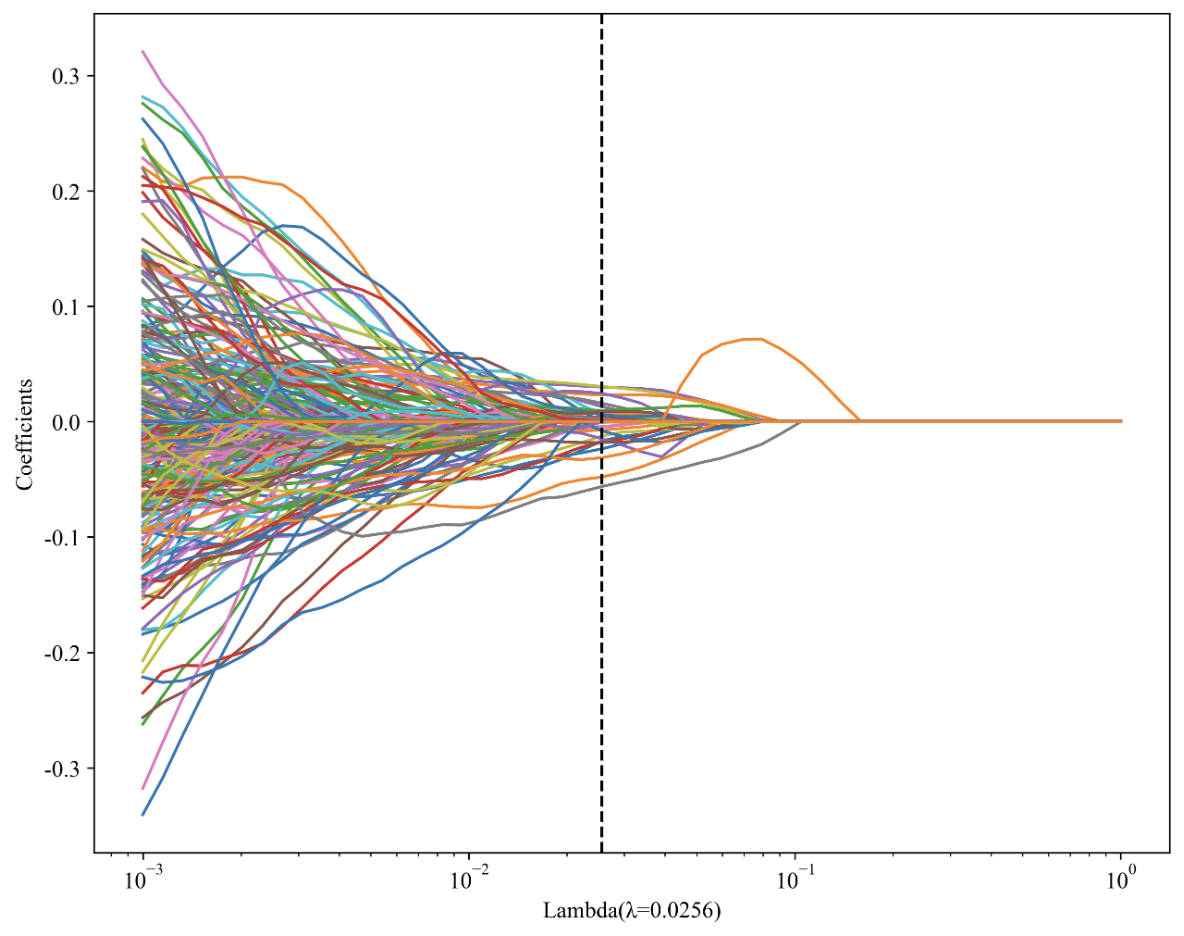

Supplement: Supplementary file 7 [file Image7.jpeg]

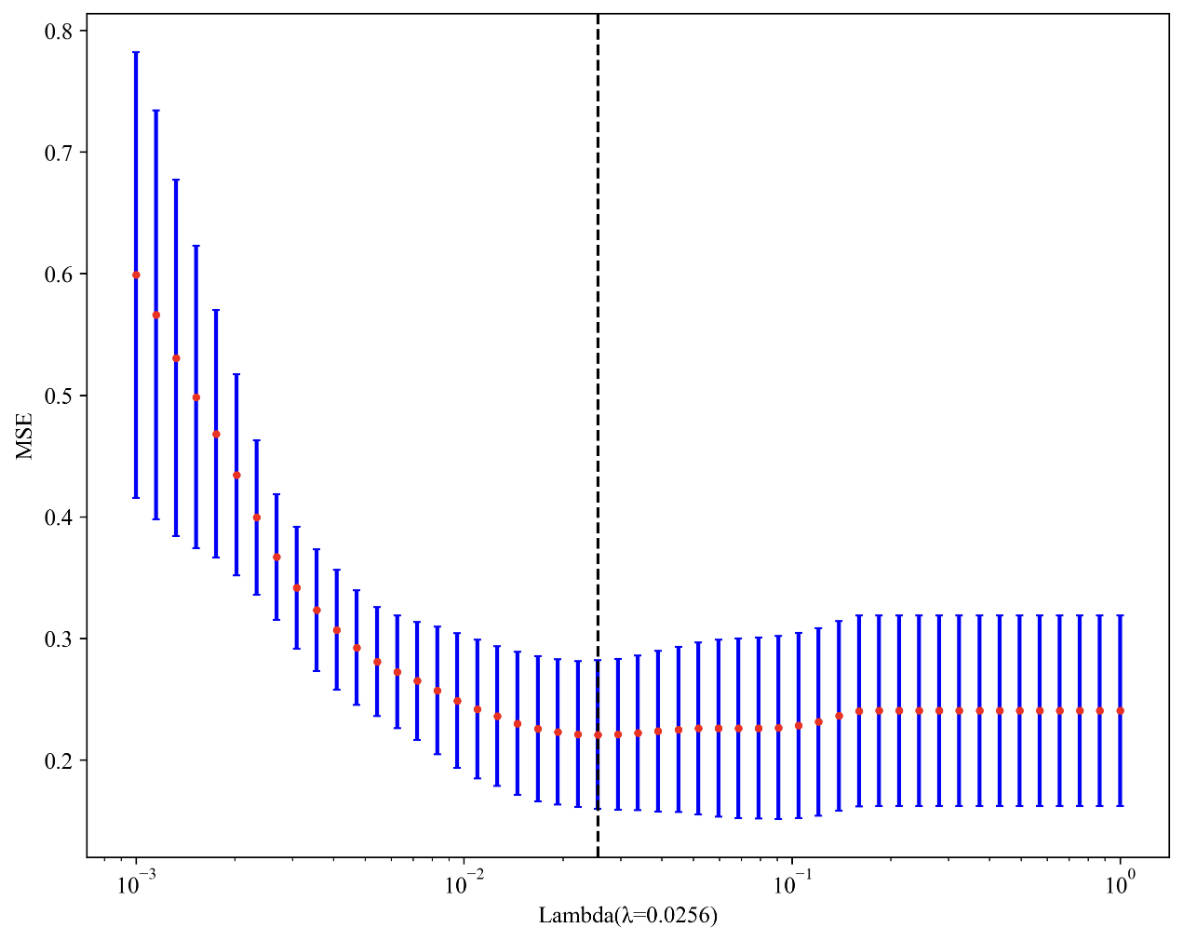

Supplement: Supplementary file 8 [file Image8.jpeg]

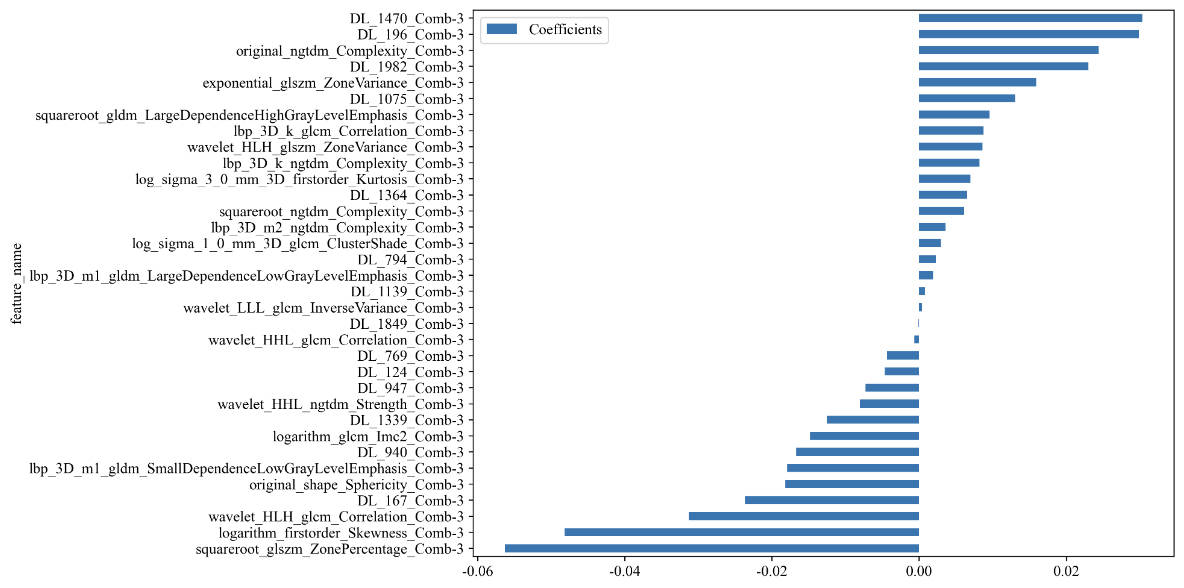

Supplement: Supplementary file 9 [file Image9.jpeg]

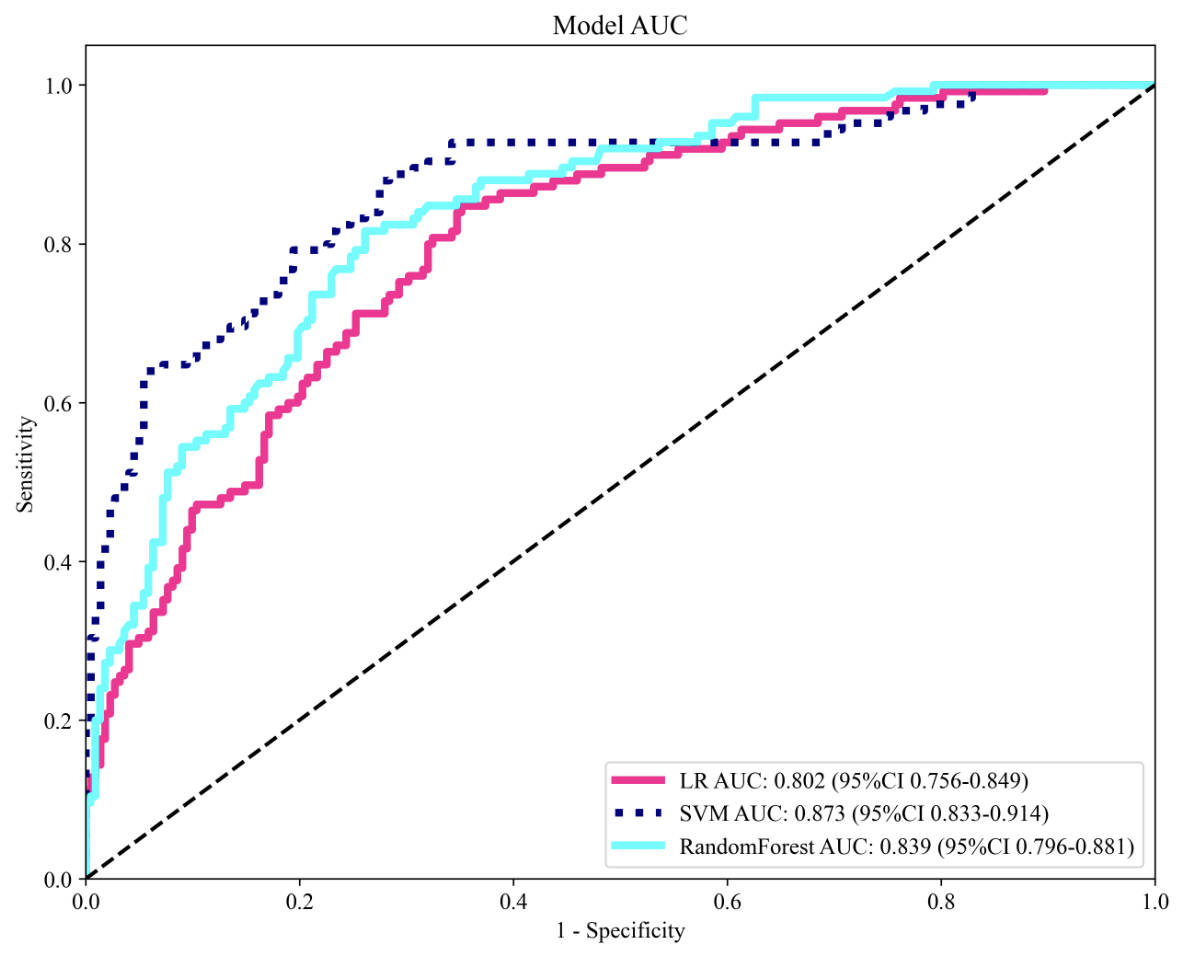

Supplement: Supplementary file 10 [file Image10.jpeg]

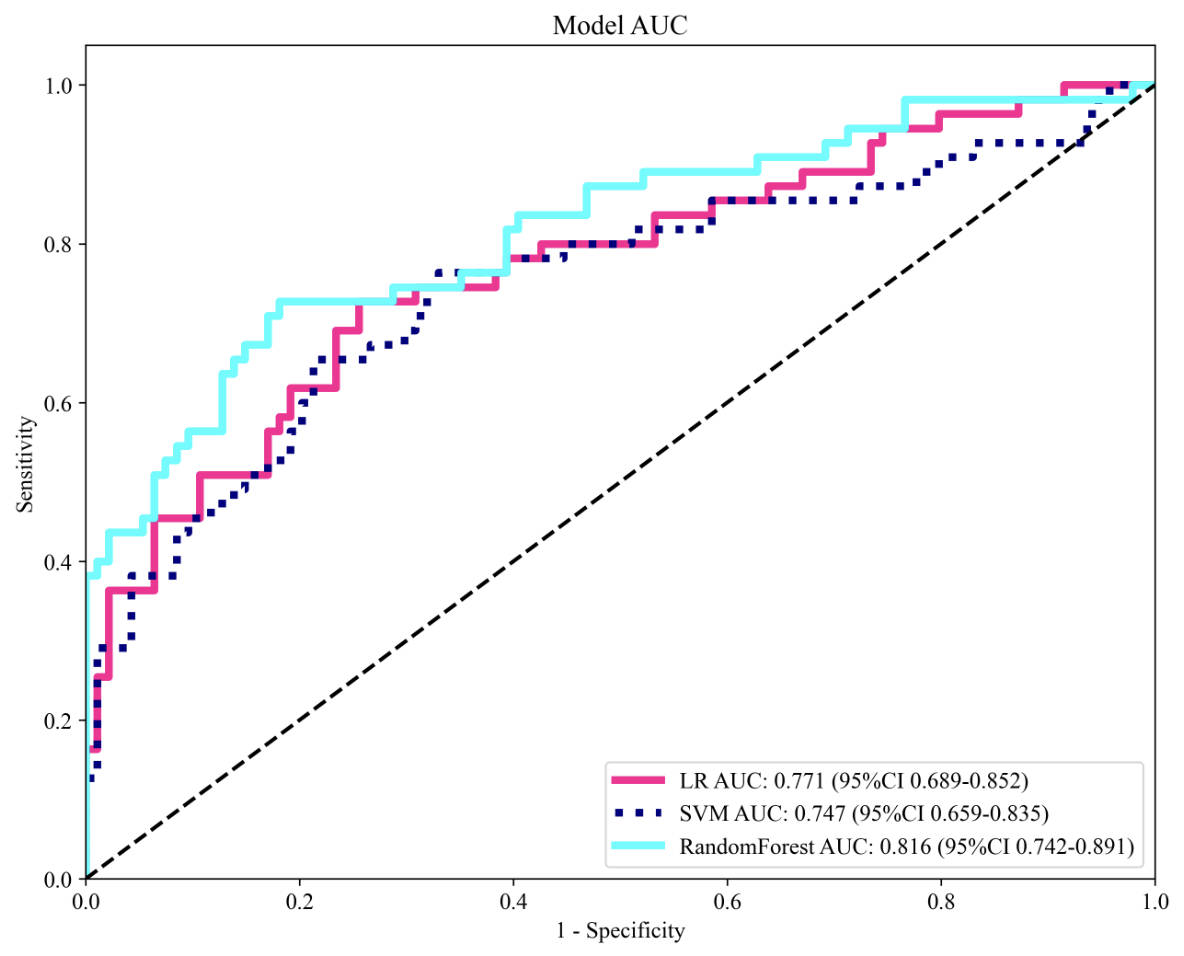

Supplement: Supplementary file 11 [file Image11.jpeg]

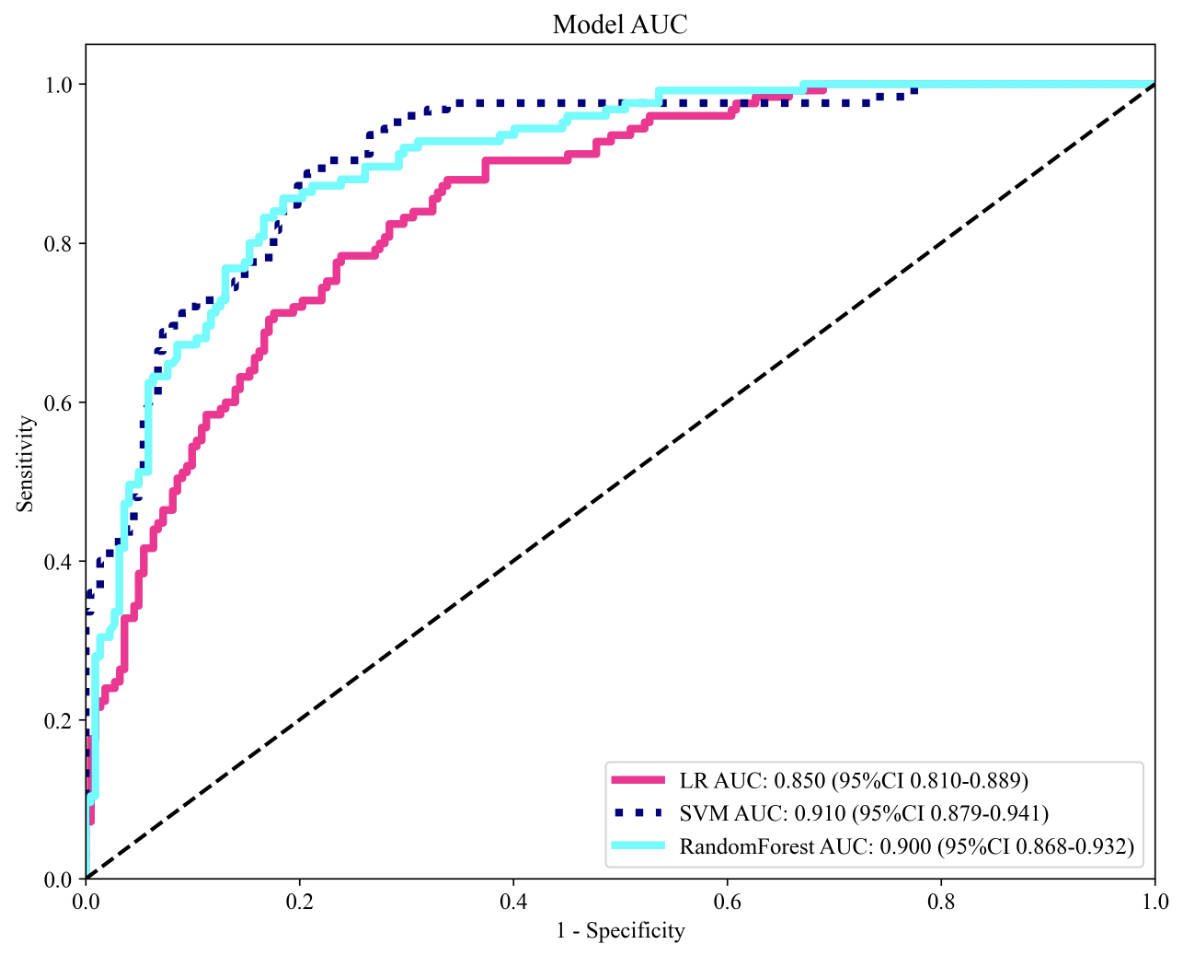

Supplement: Supplementary file 12 [file Image12.jpeg]

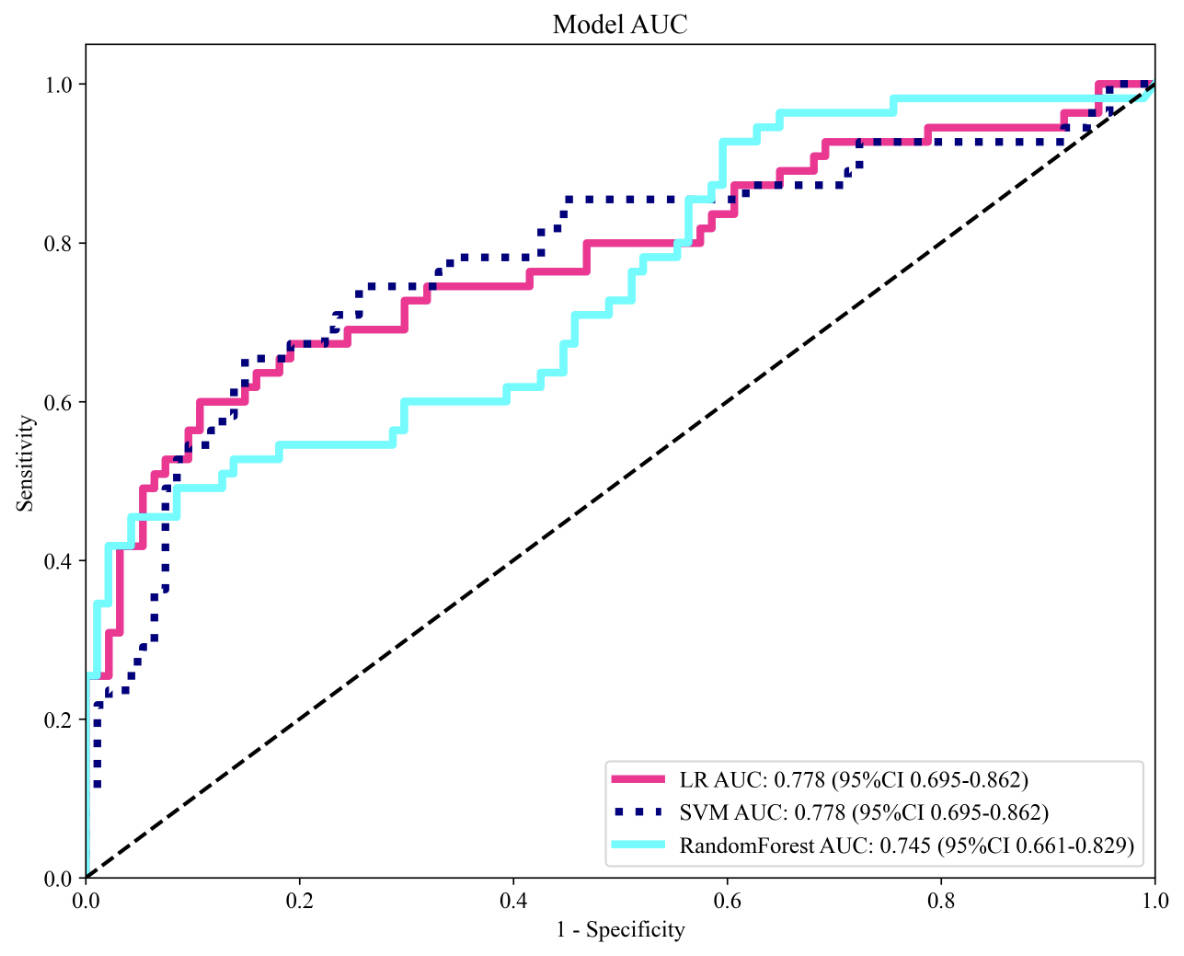

Supplement: Supplementary file 13 [file Image13.jpeg]

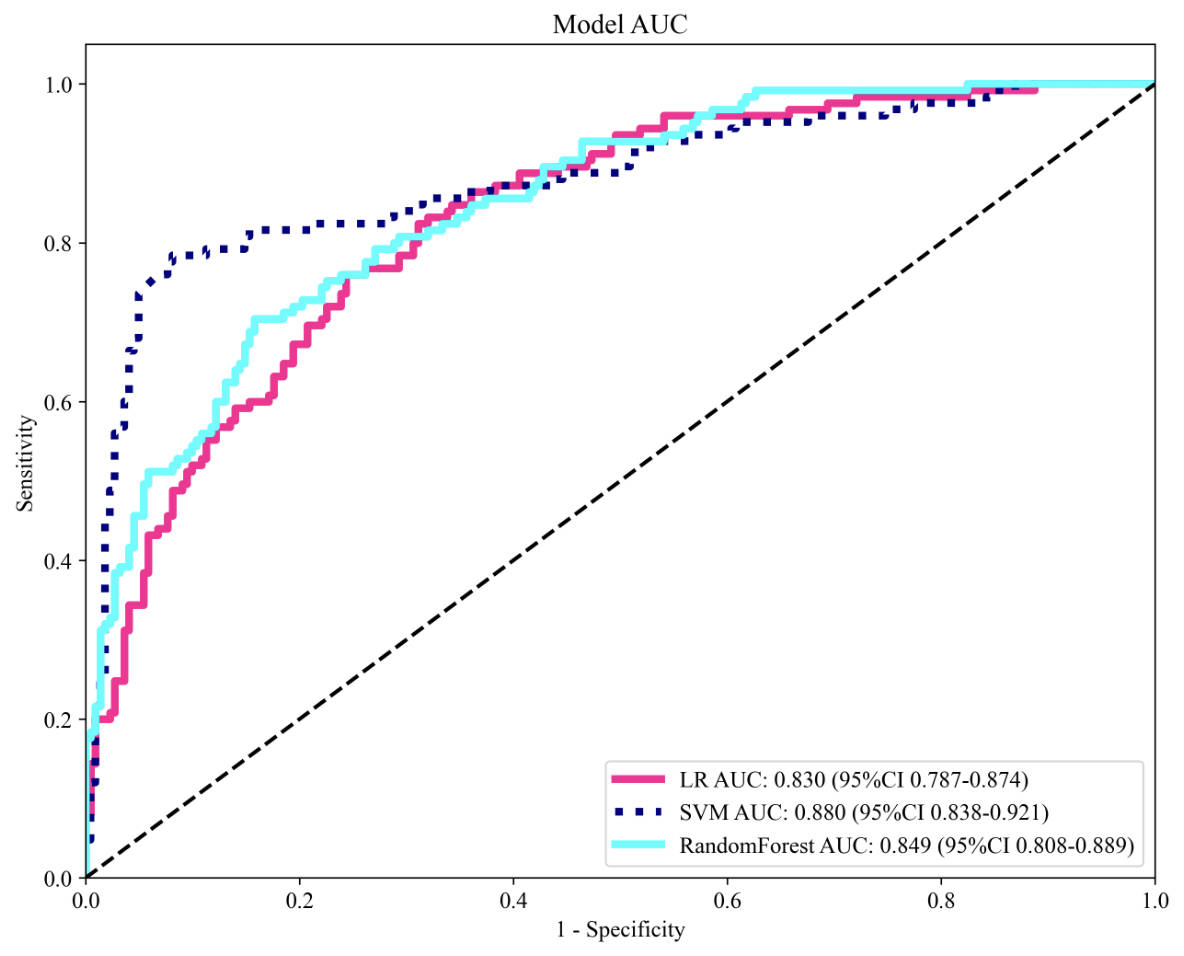

Supplement: Supplementary file 14 [file Image14.jpeg]

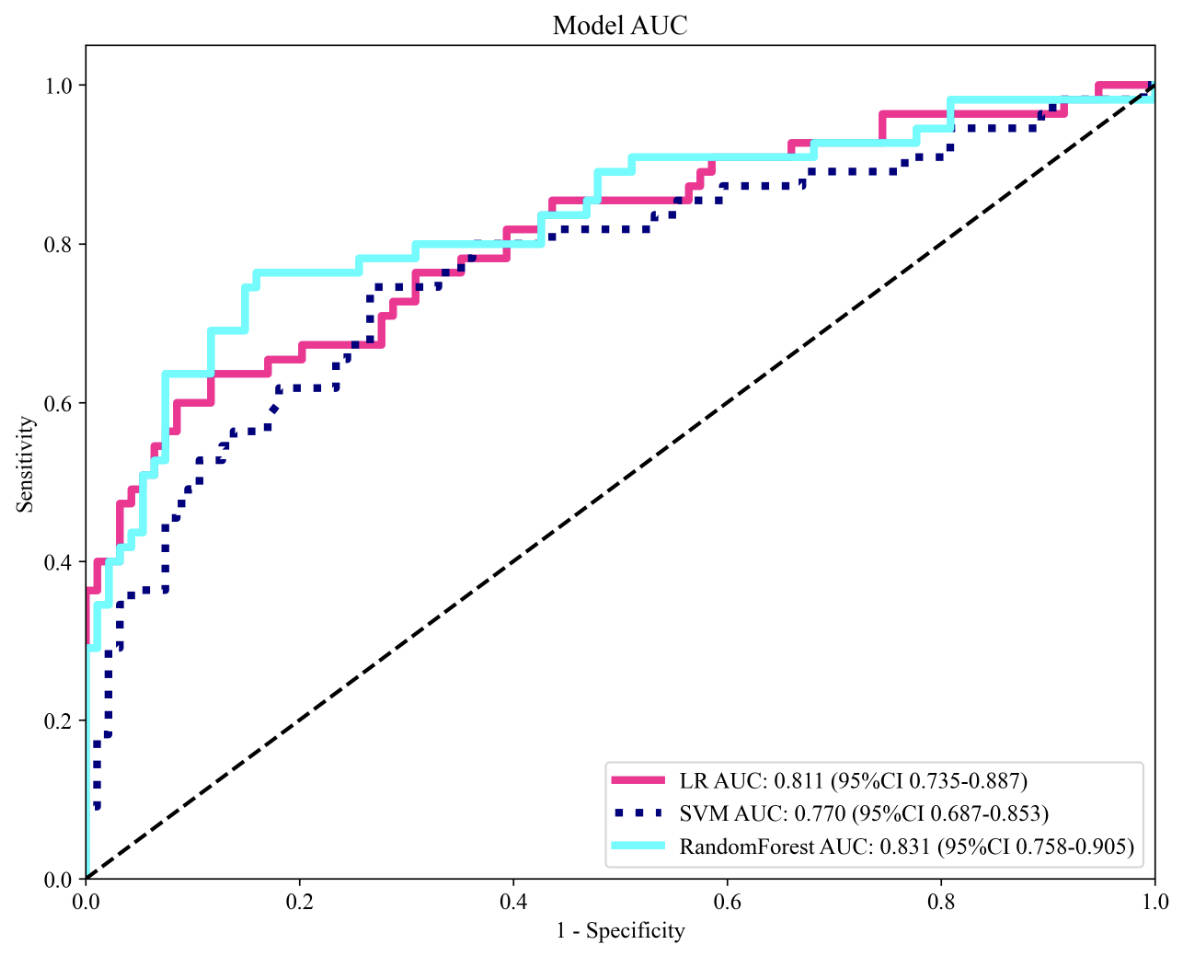

Supplement: Supplementary file 15 [file Image15.jpeg]
