# Supplementary material for: Multi-channel multiphase CT-based deep learning and radiomics fusion model for noninvasive pathological grading of clear cell renal cell carcinoma
Source: Front Oncol. 2026 Jan 15;15:1710329. doi: 10.3389/fonc.2025.1710329 (PMC12851889; doi:10.3389/fonc.2025.1710329)
Supplement: Supplementary file 16 [file Table1.docx]

| comb-1 | model_name | Accuracy | AUC | 95% CI | Sensitivity | Specificity | PPV | NPV | Task |
| --- | --- | --- | --- | --- | --- | --- | --- | --- | --- |
|  | LR | 0.72 | 0.802 | 0.7557 - 0.8492 | 0.848 | 0.649 | 0.576 | 0.883 | train |
|  | LR | 0.738 | 0.771 | 0.6894 - 0.8522 | 0.727 | 0.745 | 0.625 | 0.824 | test |
|  | SVM | 0.781 | 0.873 | 0.8326 - 0.9136 | 0.88 | 0.725 | 0.643 | 0.915 | train |
|  | SVM | 0.738 | 0.747 | 0.6591 - 0.8345 | 0.655 | 0.787 | 0.643 | 0.796 | test |
|  | RandomForest | 0.767 | 0.839 | 0.7965 - 0.8806 | 0.816 | 0.739 | 0.637 | 0.877 | train |
|  | RandomForest | 0.785 | 0.816 | 0.7421 - 0.8908 | 0.727 | 0.819 | 0.702 | 0.837 | test |
| comb-2 | model_name | Accuracy | AUC | 95% CI | Sensitivity | Specificity | PPV | NPV | Task |
|  | LR | 0.769 | 0.850 | 0.8096 - 0.8894 | 0.784 | 0.761 | 0.649 | 0.862 | train |
|  | LR | 0.785 | 0.778 | 0.6951 - 0.8616 | 0.6 | 0.894 | 0.767 | 0.792 | test |
|  | SVM | 0.824 | 0.910 | 0.8790 - 0.9411 | 0.904 | 0.779 | 0.698 | 0.935 | train |
|  | SVM | 0.779 | 0.778 | 0.6946 - 0.8616 | 0.655 | 0.851 | 0.720 | 0.808 | test |
|  | RandomForest | 0.83 | 0.900 | 0.8679 - 0.9322 | 0.856 | 0.815 | 0.723 | 0.910 | train |
|  | RandomForest | 0.772 | 0.745 | 0.6613 - 0.8286 | 0.455 | 0.957 | 0.862 | 0.750 | test |
| comb-3 | model_name | Accuracy | AUC | 95% CI | Sensitivity | Specificity | PPV | NPV | Task |
|  | LR | 0.758 | 0.830 | 0.7870 - 0.8736 | 0.76 | 0.757 | 0.638 | 0.848 | train |
|  | LR | 0.792 | 0.811 | 0.7347 - 0.8866 | 0.636 | 0.883 | 0.761 | 0.806 | test |
|  | SVM | 0.87 | 0.880 | 0.8384 - 0.9210 | 0.784 | 0.919 | 0.845 | 0.883 | train |
|  | SVM | 0.738 | 0.770 | 0.6866 - 0.8532 | 0.745 | 0.734 | 0.621 | 0.831 | test |
|  | RandomForest | 0.793 | 0.849 | 0.8079 - 0.8892 | 0.704 | 0.842 | 0.715 | 0.835 | train |
|  | RandomForest | 0.812 | 0.831 | 0.7577 - 0.9049 | 0.764 | 0.840 | 0.737 | 0.859 | test |
